# Changes in Lipid Metabolites and Enzyme Activities of Wheat Flour during Maturation

**DOI:** 10.3390/foods13162537

**Published:** 2024-08-14

**Authors:** Yanyan Chen, Yingtao Yu, Xin An, Huihui Zhang, Wei Gong, Ying Liang, Jinshui Wang

**Affiliations:** College of Biological Engineering, Henan University of Technology, Zhengzhou 450001, China; 18336073682@163.com (Y.C.); 2023910004@stu.haut.edu.cn (Y.Y.); graceax@163.com (X.A.); 13037506338@163.com (H.Z.); gw1754163531@163.com (W.G.); waitliangying@foxmail.com (Y.L.)

**Keywords:** wheat flour maturation, lipid metabolites, oxidative dynamics, enzymatic activities

## Abstract

The maturation of wheat flour is a transformative process that elevates its processing and culinary attributes to their peak performance levels. Despite extensive research on starch and gluten protein modifications, the impact of lipid changes has been largely unexplored. This study addresses this gap by examining the maturation of freshly milled wheat flour at 15 °C, 25 °C, and 40 °C over 60 days, focusing on enzymatic activities—lipase, lipoxidase, and catalase—and lipid metabolites, including free fatty acids, conjugated trienes, *p*-anisidine value, and total oxidation value. The results of this study showed that free fatty acids continued to increase at all temperatures, with the most significant increase of 50% at 15 °C. The *p*-anisidine value followed a pattern of initial increase followed by a decline, while conjugated trienes were markedly higher at 40 °C, suggesting temperature’s significant influence on lipid peroxidation. Notably, total oxidation values became erratic post 30 days, indicating a shift in oxidative dynamics. This study underscores the correlation between lipid metabolites and enzymatic activities, revealing the enzymes’ pivotal role in lipid oxidation. The interplay of temperature and time offers valuable insights for optimizing wheat flour maturation, ensuring superior quality for various applications.

## 1. Introduction

Wheat is one of the world’s primary food crops, providing sustenance to billions of people globally. Wheat can be processed into a variety of food products, such as bread, pasta, cookies, and buns. The maturation process of wheat flour significantly influences the quality of these products, with various factors accelerating or inhibiting flour aging [1]. Wheat flour maturation is a multifactorial process, with time being a critical factor. Allowing flour to mature enhances its characteristics, while optimal temperature and humidity levels facilitate this process. The flour’s composition—whether it is pure wheat or a blend of grains—also influences maturation conditions. Although some modern methods may incorporate enzymes or chemicals to modulate ripening, traditional approaches typically avoid the use of such additives. The wheat flour maturation process is a critical stage in the production of high-quality wheat flour and is generally practiced across the wheat flour production industry. While controlling the temperature and time during the wheat flour maturation process is beneficial for enhancing the quality of flour for baking, it also presents certain challenges. These include the complexity of precise control, potential increased costs due to the need for sophisticated equipment, and the risk of over-maturation if the process is not carefully monitored. Additionally, there may be limitations in flexibility and potential environmental impacts due to energy consumption. The dependency on technology and the need for skilled labor to operate and maintain the equipment can also be considered disadvantages. In a pioneering study by Lagrain et al. [2], the authors compared the performance of matured and non-matured wheat flour in bread production. They found that the use of matured flour significantly enhanced the bread’s crumb structure and extended its shelf life, providing valuable insights into the benefits of the maturation process for the baking industry. When wheat stocks are low, freshly harvested wheat is processed almost immediately, posing challenges for millers and bakers. Conversely, freshly milled wheat flour, often referred to as “immature”, produces dough that is less desirable compared to properly aged flour. Aging involves chemical and physical changes that enhance dough resistance to mixing, gas retention, and baking volume [3,4]. Newly harvested wheat flour undergoes changes in proteins, starches, and lipids during maturation, affecting its overall quality. Factors such as temperature, humidity, oxygen content, light intensity, and maturation time influence the flour maturation process, thereby allowing control over flour aging [5]. Studies indicate that storing tender flour for more than one to two months improves its quality, a phenomenon linked to storage conditions such as temperature and humidity [6]. Cenkowski et al. [7] demonstrated that the quality of flour produced by modern milling techniques improves significantly after one or two months of storage, primarily due to changes in moisture content and storage temperature. During the maturation of wheat flour, gluten proteins undergo significant transformations, leading to the development of a more elastic and extensible gluten network. This enhancement results in baked goods that boast an improved crumb structure, increased chewiness, and a more uniform texture. The maturation process, when combined with intensified baking conditions, augments the flour’s gas retention capacity. This improvement translates into the production of bread and other baked goods with superior volume, height, and porosity. Furthermore, oxidation reactions play a crucial role in reinforcing the gluten structure, while the interactions between proteins and lipids refine dough handling and baking attributes. However, it is essential to strike a balance in the maturation period [8]. Studies indicate that over-aging can diminish the gluten strength of wheat flour. As gluten is responsible for providing structure and elasticity to baked products, any reduction in its strength can lead to a less chewy texture and an inferior mouthfeel. In addition to textural changes, the flavor and aroma profiles of wheat flour may evolve during the aging process, which can significantly impact the sensory perception of the final baked goods. These alterations can potentially affect the overall appeal to consumers, making it imperative to optimize the maturation duration to preserve the desirable qualities of wheat flour [9].

Fat, one of the three major nutrients, is present in small amounts in wheat. However, during the maturation process, the oxidative degradation of fats in the presence of oxygen deteriorates wheat flour quality [10]. Lipid oxidation impacts flour’s processing quality, organoleptic properties, and nutritional value. This oxidation can occur via enzymatic pathways or auto-oxidation, both of which influence the stability of whole meal flour. During the maturation process, lipase in flour releases free fatty acids, which are then oxidized by lipoxygenase or through auto-oxidation [11]. Lipase hydrolyzes fats to free fatty acids, which form hydroperoxides and alcohols through lipoxygenase action, leading to the production of volatile compounds, such as aldehydes, ketones, and alcohols. Lipid oxidation in food is a complex chain reaction that initially produces primary products (peroxides) and, over extended oxidation periods, generates secondary oxidation products, resulting in undesirable sensory and biological effects [12]. Free fatty acids can indicate hydrolytic rancidity of triglycerides through enzymatic or spontaneous hydrolysis [13]. The *p*-anisidine value is a useful method for evaluating lipid oxidation in fats and oils, reflecting the formation of aldehyde–carbon bonds during secondary lipid oxidation [14]. The variation at K268 nm, used to detect secondary oxidation, differs from *p*-anisidine analysis as no ketones are detected. The total oxidation value (TOTOX), which includes both primary and secondary oxidation assessments, provides a comprehensive measure of the oxidation state of fats and oils [15].

While there is a growing interest in the field, existing studies on lipid changes during wheat flour maturation have been limited in scope, often focusing on isolated aspects of the maturation process without a systematic and comprehensive analysis [16,17], Narisawa et al. [16] only investigated the effect of lipoxygenase on the formation of volatile chemicals in the dough. Devi et al. [18] only studied the effect of lipids in wheat flour on the paste and texture characteristics of flour and did not find the reason for the deep lipid oxidation.

Currently, studies on lipid changes during wheat flour maturation are limited in scope and lack systematic and comprehensive analysis. This study investigates the interaction between temperature and time and its effect on the primary chemical components of wheat flour. By examining changes in lipid metabolites—free fatty acids (FAAs), conjugated trienes (K268 nm), *p*-anisidine value (P-AV), and total oxidation value (TOTOX)—and enzyme activity during wheat flour maturation, this research aims to provide a theoretical basis for optimizing the maturation and processing of wheat flour.

## 2. Materials and Methods

### 2.1. Wheat Preparation and Maturation

The wheat used in this study was obtained from the new Bainong 307 variety harvested in June 2023 from Xinxiang City, China. Wheat flour was obtained by grinding the particles in a cyclone mill (Tronic, Hangzhou, China). The samples were stored for 60 days at different temperature conditions, i.e., 15 ± 1 °C, 25 ± 1 °C, and 40 ± 1 °C. Wheat samples were collected regularly every 10 days throughout the ripening period and were immediately experimented with and placed in a 4 °C refrigerator for a short time. The flour was not bleached and did not contain any added ingredients. All of the chemicals used in the analytical procedures were of the highest purity grade.

Lipid extraction: 3 times the volume of petroleum ether was added to the sample and extracted for more than 12 h; the upper solution was taken and filtered, the solvent was rotary evaporated, and about 3 mL of the remaining solution was put into a 40 °C oven to dry the remaining solvent and obtain the oil. The extracted fats and oils were stored in a −80 °C refrigerator.

### 2.2. Measurement of Free Fatty Acids (FFA)

The free fatty acid content was determined according to the method described by Goffman et al. [19]. Then, 0, 15, 20, 30, 40, and 50 μmol/L captanoic acid concentrations were dissolved in 5 mL iso-octane solution and 1.0 mL Cu-pyridine acetate was added. The standard curve was obtained by measuring the absorbance at 715 nm. In brief, 3 mL of iso-octane was added to 100 mg of the sample. The mixture was shaken at 250 rpm for 5 min and then centrifuged at 4000 rpm for 5 min. Subsequently, 2 mL of the supernatant was combined with 1 mL of an aqueous solution containing 3% (*v*/*v*) pyridine and 5% (*w*/*v*) copper acetate. The mixture was shaken at 250 rpm for 5 min, and the absorbance of the iso-octane phase was measured at 715 nm (UV-Vis Spectrophotometer (P4): Shanghai Mepda Instrument Co., Shanghai, China). Each sample group was analyzed at least four times in parallel, and the average value was reported.

### 2.3. Conjugated Triene Value (K268 nm)

The conjugated triene value was measured at 268 nm according to ISO3656:2011 [20]. Specifically, 0.1 g of oil was placed in a 25 mL volumetric flask and dissolved in iso-octane. A 1 mL aliquot of this solution was then diluted with 4 mL of iso-octane, and the absorbance was measured at 268 nm to assess oxidation products. Iso-octane was used as a blank.

### 2.4. p-Anisidine Value (P-AV)

The *p*-anisidine value (P-AV) was determined according to ISO 6885:2016 [21]. An oil sample (1 mg) was dissolved in a 25 mL volumetric flask and diluted with iso-octane. A 5 mL aliquot of this solution was thoroughly mixed with 1 mL of *p*-anisidine solution. The mixture was allowed to react in the dark for 10 min, and the absorbance was recorded at 350 nm using a spectrophotometer.

### 2.5. Total Oxidation Value (TOTOX)

The method of Jensen et al. [22] was referred to and slightly improved. Take 1.0 g of wheat flour, add 5 mL of trichloromethane–methanol (7:3, *v*/*v*), shake for 2 min, and immediately centrifuge to take all the supernatant in a 10 mL colorimetric tube trichloromethane–methanol (7:3, *v*/*v*) to fix the volume. Add 50 μL of aqueous ferrous chloride tetrahydrate (3.5 g/L) and aqueous potassium thiocyanate (300 g/L), respectively, shake well, and then place in the dark for 5 min. Measure the absorbance at 500 nm, and then bring it into the standard curve to calculate the quantification, and make a blank solution for reference at the same time.

The total oxidation status, encompassing primary and secondary oxidation products, was assessed by calculating the TOTOX value using the formula provided by Chew et al. [23]:

TOTOX = P-AV + 2 × PV(1)

where P-AV is the *p*-anisidine value and PV is the peroxide value.

### 2.6. Lipase Activity Assay

Lipase activity was measured following the method provided by Jia et al. with modifications [24]. For enzyme extraction, 1.0 g of the sample was dissolved in 5 mL of Tris-HCl buffer (0.05 mol/L, pH 8.0), shaken, and incubated in an ice bath at 4 °C for 30 min. The mixture was then centrifuged at 10,000× *g* for 10 min at 4 °C, and the supernatant was collected. The substrate used was pnpc-acetonitrile (10 mmol/L, pH 8.0). The reaction system included 20 μL of pnpc-acetonitrile, 1780 μL of Tris-HCl buffer, and 200 μL of enzyme extract at 37 °C. Absorbance values were recorded at 405 nm at 30 s intervals for 3 min. One unit of lipase activity was defined as an increase of 0.01 in absorbance per minute. Each sample group was analyzed at least four times in parallel, and the average value was reported.

### 2.7. Lipoxygenase Activity Assay (LOX)

Lipoxygenase activity was measured according to the method provided by Liu et al. with modifications [25]. For enzyme extraction, 1.0 g of the sample was dissolved in 5 mL of PBS (0.1 mol/L, pH 7.5), shaken, and incubated in an ice bath at 4 °C for 30 min. The mixture was centrifuged at 10,000× *g* for 10 min at 4 °C, and the supernatant was collected. The substrate, linoleic acid, was prepared by dissolving 0.5 mL of Tween-20 in 10 mL of boric acid buffer (0.05 mol/L, pH 9.0), then 0.5 mL of linoleic acid was added, shaken well, and the pH was adjusted to 7.0. The reaction system included 2.89 mL of sodium acetate buffer, 90 μL of linoleic acid substrate, and 20 μL of enzyme extract. An increase of 0.01 in absorbance per minute was defined as a unit of LOX activity. Each sample group was measured at least four times in parallel, and the average value was reported.

### 2.8. Catalase Activity Measurement (CAT)

Catalase activity (u/g) was measured using a CAT kit (Kit No. BC0205, Beijing Solarbio, Beijing, China), with about 0.1 g tissue weighed and 1 mL extract added for ice bath homogenization. Centrifuge 8000× *g* at 4 °C for 10 min, take the supernatant, and put it on ice for the test. Before the test, the CAT test solution was immersed in a water bath at 25 °C for more than 10 min. Then, 10 μL of the sample and 190 μL of the working liquid were added into the micro quartz colorimeter, immediately mixed, and timed, and the initial absorption value A1 at 240 nm and the absorption value A2 after 1 min were recorded. Calculate ΔA = A1 − A2, CAT activity was ΔA/0.1 g.

### 2.9. Statistical Analysis

All of the experiments were repeated at least three times, and mean values were reported. The significance of differences was analyzed using Duncan’s Multiple Comparison with SPSS 27 software at a significance level of *p* < 0.05. Graphs were generated using Origin 2021 software. The correlation between the 19 samples was determined using the Pearson correlation coefficient method (*p* ≤ 0.05 (*) and *p* ≤ 0.01 (**)).

## 3. Results and Discussion

### 3.1. Free Fatty Acid Variation during Wheat Flour Maturation

**Figure 1:**
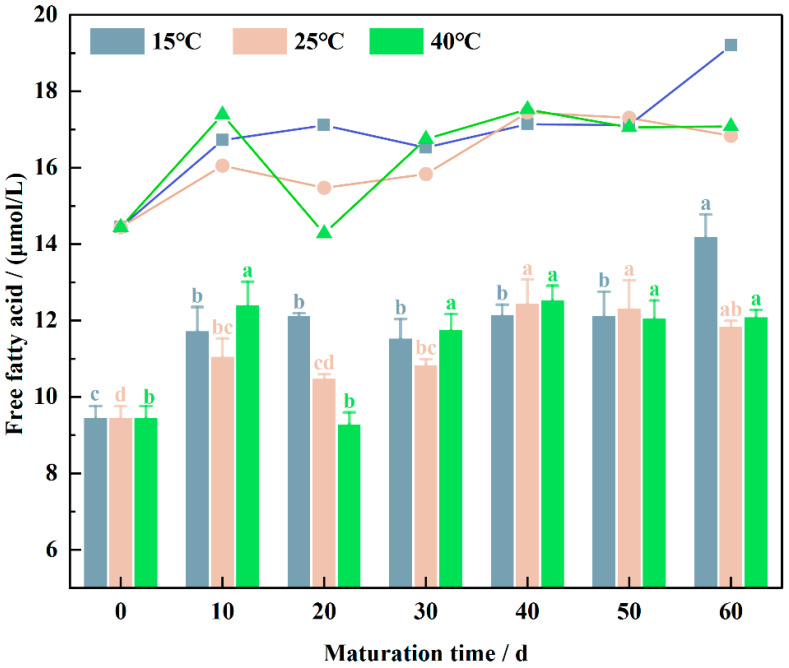
Change in free fatty acid content of wheat flour samples in the maturation process, the lowercase letters indicate significant differences in the same sample at different maturation times at *p* < 0.05.

Free fatty acids (FFAs) are products of fat hydrolysis, and their levels indicate the extent of fat hydrolysis during the maturation of wheat flour [13]. As illustrated in Figure 1, the FFA content in the control wheat flour was 9.44 μmol/L. The free fatty acid content increased significantly (*p* < 0.05) after maturation compared to the control. This is in agreement with the study conducted by Tian et al. [26]. The FFA content increased gradually during the early stages of wheat maturation. Upon maturation at 15 °C, the FFA content in wheat flour increased and continued to rise gradually throughout the maturation period, except for a slight decrease observed at 30 days. At 25 °C and 40 °C, the FFA content exhibited an M-shaped trend: it increased from 0 to 10 days, decreased from 10 to 20 days, increased gradually from 20 to 40 days, and then decreased from 40 to 60 days, with notable fluctuations occurring around 20 days of maturation.

This dynamic change in FFA content during wheat flour maturation can be attributed to two main factors. Firstly, lipase activity leads to the hydrolysis of glycerides, resulting in the production of free fatty acids [27]. Secondly, these free fatty acids are subject to oxidation by lipoxygenase, producing various metabolites [28]. These concurrent processes result in the dynamic variation of FFA levels. During the early stages of maturation (0–30 days), high levels of oxidation lead to a decrease in FFA content. In the later stages of maturation (40–60 days), the FFA content stabilizes. The decline in FFA content during the early stages of maturation is a significant factor contributing to the reduction in the quality of matured wheat flour [29].

### 3.2. Conjugated Triene Levels in the Maturation of Wheat Flour

**Figure 2:**
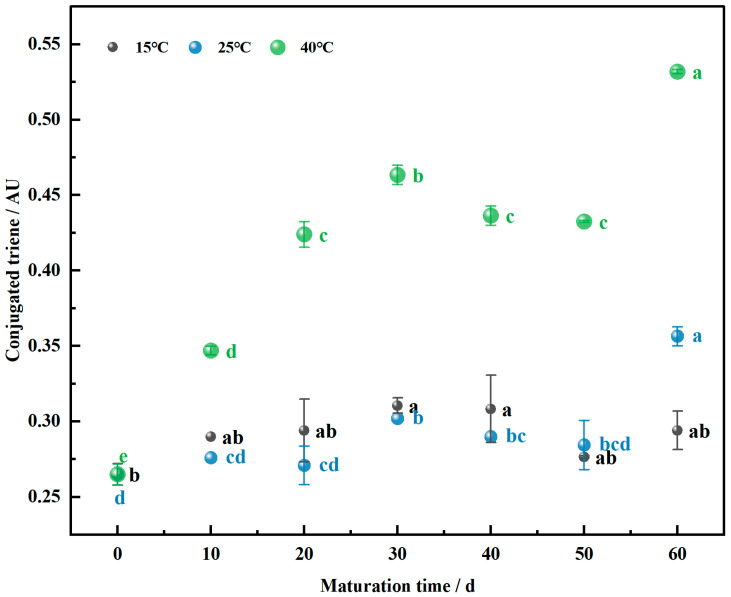
Changing the conjugated triene value of wheat flour samples during the maturation process, the lowercase letters denote significant differences between maturation times at the same maturation temperature at *p* < 0.05.

During the maturation of wheat flour, unsaturated fatty acids undergo oxidation to form peroxides, which further decompose to generate free radicals. These free radicals can react with double bonds in other fatty acids, leading to the formation of conjugated trienes [30,31]. As depicted in Figure 2, the conjugated triene values of wheat flour at all three maturation temperatures significantly increased (*p* < 0.05) with prolonged maturation time. Additionally, the conjugated triene values progressively increased with rising maturation temperatures.

Komeine et al. [32] found that the conjugated triene values of matured cereal flours were significantly higher than those of fresh cereal flours during the maturation process. This finding underscores the impact of maturation on the oxidative state of flour. The pattern of conjugated triene value changes at 25 °C and 40 °C followed a similar trend: an increase from 0 to 30 days, a significant decrease at 40 days (*p* < 0.05), and a subsequent increase from 40 to 60 days. In contrast, at 15 °C, the conjugated triene values showed less variation over time, with a notable decrease observed at 50 days (*p* < 0.05). This trend suggests that at the lower temperature of 15 °C, the rate of lipid oxidation in wheat flour is reduced. As the oxidation level increases with higher temperatures, the content of conjugated trienes also rises, indicating a positive correlation between temperature and conjugated triene values within a certain range.

As maturation time increases, the rate of lipid oxidation reactions in wheat flour slows down, resulting in the decreased production of free radicals and peroxides. Consequently, the decomposition of conjugated trienes also slows, leading to reduced conjugated triene content. This decrease can be attributed to the increased unsaturation of unsaturated fatty acids and the formation of ketones over time [33]. The smaller increase in conjugated triene content during the later stages of lipid oxidation indicates a decline in wheat flour quality with extended maturation time.

### 3.3. p-Anisidine Values Shift in the Wheat Flour Maturation Process

Unsaturated fatty acids in fats and oils, upon oxidation, generate hydroperoxides, which are unstable and prone to further oxidation and decomposition. This process results in the formation of α- or β-unsaturated aldehydes and other secondary oxidation products, with aldehydes constituting approximately half of the oxidized volatiles of fats and oils. The *p*-anisidine value (P-AV) measures the aldehyde content in these secondary oxidation products, reflecting the degree of oxidative rancidity in fats and oils [34].

**Figure 3:**
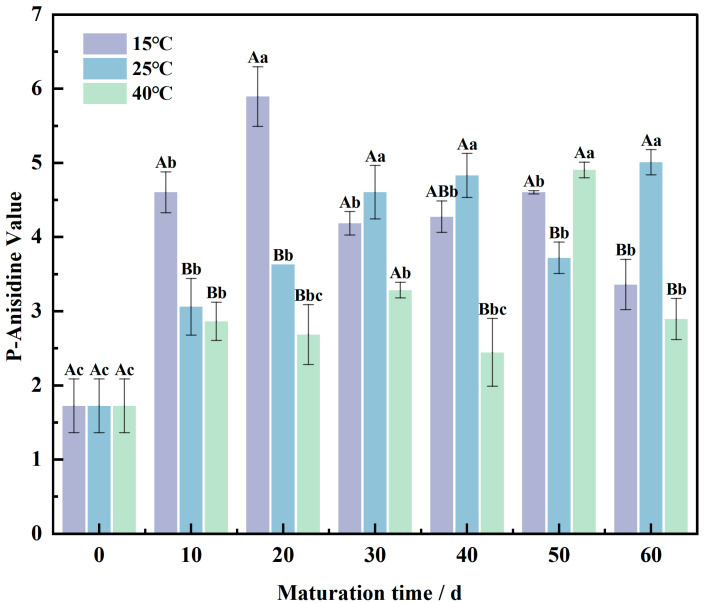
Changing of *p*-anisidine value in wheat flour samples during the maturation process, the lowercase letters indicate significant differences between maturation times at the same maturation temperature, and uppercase letters indicate significant differences between maturation temperatures at the same maturation time (*p* < 0.05).

As shown in Figure 3, the P-AV of wheat flour exhibited a trend of an initial increase followed by a decrease under various temperature conditions during maturation. At 15 °C, the P-AV peaked at 5.90 after 20 days, followed by a significant decline at 30 days (*p* < 0.05). From 30 to 60 days, the P-AV remained relatively stable, reaching a minimum of 3.36 at 60 days. At 25 °C, the P-AV increased gradually from 0 to 40 days, then significantly decreased at 50 days (*p* < 0.05), peaking at 5.01 at 60 days. At 40 °C, the P-AV was 2.45 at 40 days, increased to 4.91 at 50 days—a 50% increase—before subsequently decreasing.

During the early stages of maturation (0–30 days), there was no significant difference in P-AV at 25 °C and 40 °C, but significant differences were observed during the later stages. At 15 °C, the P-AV was highest during the early stage of maturation, while it was lowest at 40 °C. This pattern can be attributed to the auto-oxidation of unsaturated fatty acids, leading to the formation of small molecules such as aldehydes, which increase the P-AV [35]. In the early stage of maturation, the P-AV rises due to intensifying oxidative rancidity. In the later stage, the oxidation rate stabilizes or slows down, and aldehydes are converted into other compounds, resulting in a decrease in P-AV.

The lower P-AV at 40 °C may be due to the rate of aldehyde synthesis being lower than the rate of their conversion into acids, which can damage cells and tissues and impair normal physiological functions [36].

### 3.4. Total Oxidizing Value Evolution during Wheat Flour Maturation

**Figure 4:**
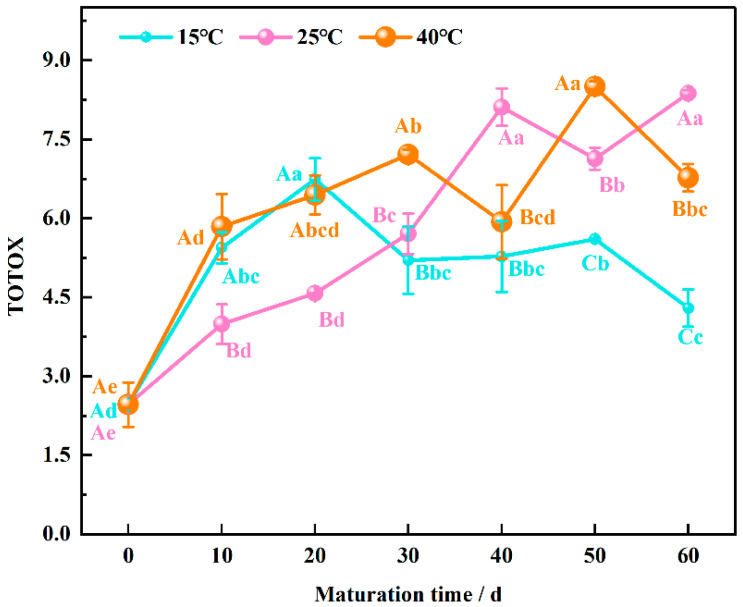
Changing of total oxidation value of wheat flour samples during the maturation process, the lowercase letters indicate significant differences between maturation times at the same maturation temperature, and uppercase letters indicate significant differences between maturation temperatures at the same maturation time (*p* < 0.05).

The total oxidation value (TOTOX) is a comprehensive indicator used to assess the degree of oxidative deterioration in lipids. It combines measures of both primary oxidation products (hydroperoxides) and secondary oxidation products (unsaturated aldehydes) [37]. As illustrated in Figure 4, the initial TOTOX of immature wheat flour was 2.46. At 15 °C, the TOTOX increased rapidly, reaching 6.74 at 20 days, and then stabilized, decreasing to 4.30 at 60 days.

At 25 °C, the TOTOX showed a gradual increase, peaking at 8.11 at 40 days, followed by a decrease at 50 days, and reaching a maximum of 8.37 at 60 days. At 40 °C, the TOTOX increased from 0 to 30 days, decreased to 5.93 at 40 days, surged by 30% at 50 days, and then significantly decreased at 60 days (*p* < 0.05).

Comparing different maturation temperatures at the same maturation time, the TOTOX was lower at 25 °C than at 15 °C and 40 °C during the early maturation period, while it was lowest at 15 °C during the late maturation period. This trend may be attributed to the high level of oxidation caused by elevated enzyme activity during the early maturation stage, resulting in higher TOTOX values. At 25 °C, oxidation intermediates (e.g., hydroperoxides) may be more stable and less likely to decompose further to form secondary oxidation products, thus lowering the TOTOX. This stability could be due to slower kinetic rates of decomposition at this temperature, contributing to the observed lower TOTOX values in the early maturation period. In the late maturation stage, reduced enzyme activity leads to lower levels of unsaturated aldehydes and, consequently, a decrease in TOTOX. This decrease suggests that the rate-limiting step in oxidation may be enzyme activity, which is more pronounced at lower temperatures, such as 15 °C, where the TOTOX values were found to be the lowest.

The high TOTOX values observed in the early maturation stage are due to the presence of significant amounts of unsaturated aldehydes, which are products of intense oxidative reactions facilitated by active enzymes. As maturation progresses, enzyme activity diminishes, leading to a reduction in the formation of these aldehydes and, therefore, a decrease in TOTOX. The unsaturated aldehydes in wheat flour possess strong antioxidant properties, which contribute to a lower degree of oxidative deterioration in mature wheat flour.

### 3.5. Endogenous Lipase Activity Patterns in Wheat Flour Maturation

**Figure 5:**
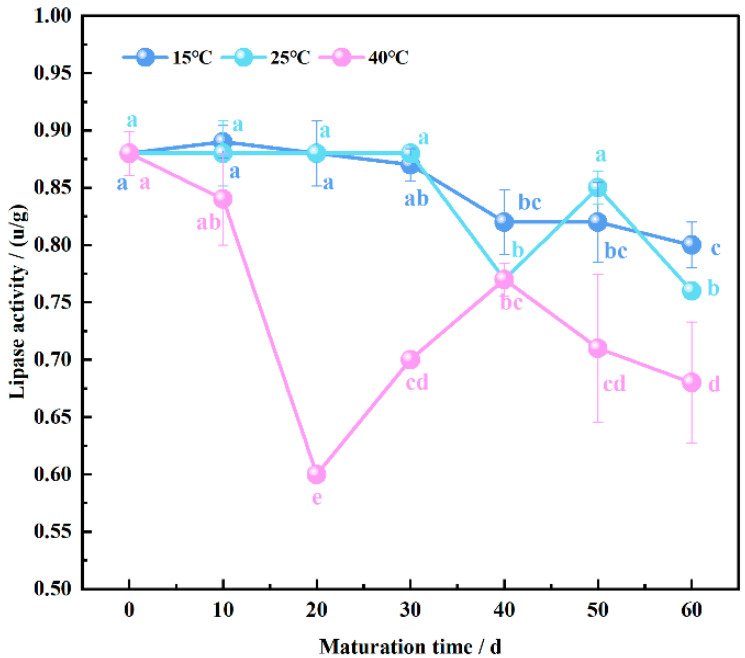
Changing pattern of endogenous lipase activity in wheat flour during maturation, the lowercase letters denote significant differences between maturation times at the same maturation temperature at *p* < 0.05.

The primary enzyme involved in lipolysis metabolism is lipase, which is generally acknowledged to regulate the rate of fat conversion [38,39]. As depicted in Figure 5, lipase activity exhibited a general decreasing trend with the extension of maturation time. At 15 °C and 25 °C, lipase activity significantly decreased at 40 days of maturation (*p* < 0.05), while at 40 °C, lipase activity decreased by 32% at 20 days, showing a significant difference (*p* < 0.05). There was no significant difference in lipase activity at 15 °C for 40–60 days of maturation, whereas at 25 °C, lipase activity displayed a tendency to initially increase and then decrease. Similarly, at 40 °C, lipase activity exhibited an initial increase followed by a decrease from 30 to 60 days of maturation.

Throughout the maturation process, lipase activity in wheat flour may undergo modulation by various factors, including temperature, humidity, oxygen concentration, and microbial presence. The temperature of 40 °C exceeds the optimum temperature for lipase activity (37 °C), leading to the accelerated denaturation of the enzyme [40]. During the early stages of maturation, wheat flour typically exhibits higher and more stable lipase activity, which may arise from endogenous factors within the wheat flour or exogenous contaminants introduced during the maturation process. However, as maturation progresses, lipase activity may gradually decline due to enzyme inactivation, degradation, or inhibition by other factors [41]. Kumar et al. [42] showed that wheat lipase, with the highest activity in flour on the 10th day of maturation, decreased further with increasing maturation time. This observation is consistent with our findings and suggests that the initial peak in activity is followed by a decline as the maturation period extends.

Nevertheless, a modest increase in lipase activity may also be observed during prolonged maturation periods. This phenomenon could result from complex chemical and biological reactions occurring during maturation, such as the alleviation of product inhibition, facilitating the neutralization of certain inhibitory factors, or enzyme activation.

### 3.6. Lipoxygenase Activity Dynamics in the Maturation of Wheat Flour

**Figure 6:**
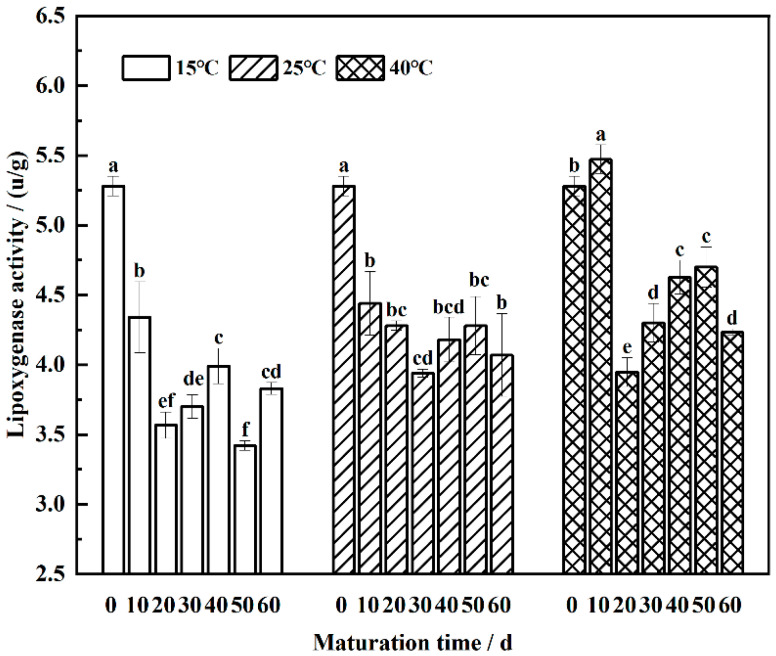
Changing pattern of endogenous lipoxygenase activity in wheat flour during maturation, the lowercase letters denote significant differences between maturation times at the same maturation temperature at *p* < 0.05.

Lipoxygenase (LOX) catalyzes the enzymatic oxidation of unsaturated fatty acids, leading to the formation of hydroperoxides [43]. As depicted in Figure 6, LOX activity exhibited an overall decreasing trend with maturation time, consistent with findings by Singh et al. [44]. Significant decreases in enzyme activity were observed from 0 to 20 days at 15 °C and from 0 to 30 days at 25 °C (*p* < 0.05). At 40 °C, LOX activity peaked at 5.27 u/g at 10 days and subsequently decreased by 28% at 20 days, demonstrating significant differences (*p* < 0.05). During the late stage of maturation, LOX activity displayed minor fluctuations.

The optimal temperature range for LOX activity is between 40 °C and 45 °C, explaining the higher enzyme activity observed at the 40 °C-maturation temperature compared to other temperatures. However, as maturation time increased, LOX activity diminished due to various factors, such as temperature fluctuations, humidity, and oxygen concentration, which can influence the enzyme’s structural integrity, thereby reducing its activity [45]. Additionally, prolonged maturation may lead to the accumulation of inhibitors that bind to the enzyme, further diminishing its activity. These inhibitors may originate from metabolic byproducts of wheat flour itself, with hydrogen peroxide, an oxidation product of free fatty acids, known to inhibit LOX activity. However, during the late stage of maturation, hydrogen peroxide is converted into secondary metabolites, potentially alleviating its inhibitory effects and consequently enhancing LOX activity.

### 3.7. Changing Pattern of Endogenous Catalase Activity in Wheat Flour

**Figure 7:**
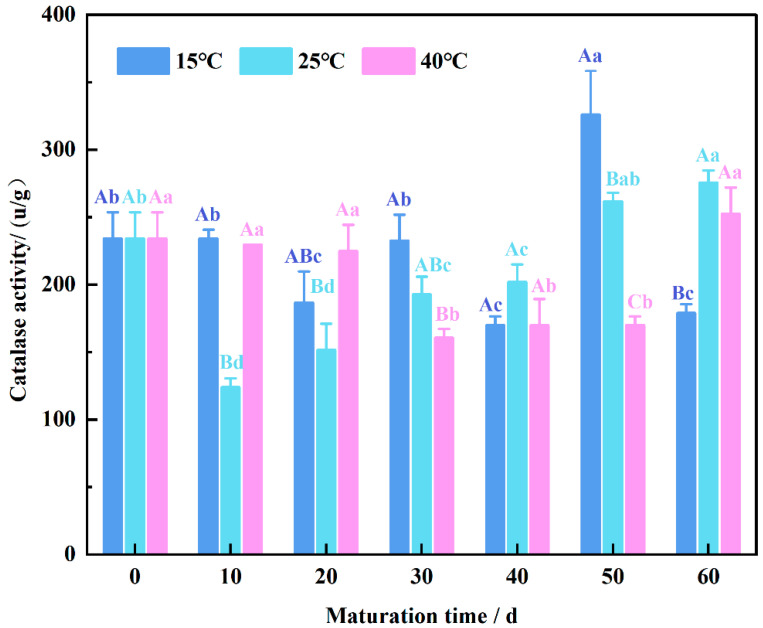
Changing pattern of endogenous catalase activity in wheat flour during maturation, the lowercase letters indicate significant differences between maturation times at the same maturation temperature, and uppercase letters indicate significant differences between maturation temperatures at the same maturation time (*p* < 0.05).

Catalase (CAT) is widely distributed in various organisms and plays a crucial role in scavenging hydrogen peroxide generated during mitochondrial electron transfer and lipid β-oxidation in plant cells, thereby protecting them from oxidative damage [46]. As illustrated in Figure 7, the initial catalase activity of immature wheat flour was recorded at 234.09 u/g. At 15 °C, enzyme activity during maturation at 20, 40, and 60 days was significantly lower than the initial activity (*p* < 0.05), except for 50 days, where activity was notably higher. At 25 °C, enzyme activity decreased slightly by 50% at 10 days of maturation, gradually increased thereafter, and surpassed the initial activity at 50 days. Within 20 days of maturation at 40 °C, no significant difference in enzyme activity was observed (*p* < 0.05), with a subsequent 31% decrease at 30 days. However, enzyme activity remained consistent between 30 and 50 days, and at 60 days, it exceeded the initial level. Yan et al. [47] showed a decreasing trend in CAT activity with maturation time, indicating the gradual deterioration of wheat.

Across the same maturation period, enzyme activity was lower at 25 °C during the pre-maturation stage and at 40 °C during the late maturation stage (*p* < 0.05). This phenomenon can be attributed to the role of catalase in decomposing hydrogen peroxide generated from lipid oxidation, thereby disrupting the chain reaction of lipid auto-oxidation and mitigating lipid oxidation processes [48]. The lower enzyme activity observed during the early maturation stage corresponds to the higher oxidation levels during this period. Conversely, as oxidation rates diminish during the late maturation stage, enzyme activity tends to rise. The sustained vitality of catalase throughout maturation indicates its pivotal role in inhibiting lipid oxidation processes.

### 3.8. Correlation Analysis of Quality Indicators

To elucidate the interplay between lipid oxidation and enzyme activity changes during the maturation process of wheat flour, correlations among various indices were examined, as presented in Table 1. Conjugated trienes exhibited a highly significant negative correlation with lipase and a significant positive correlation with total oxidation values, indicating a close association between lipase activity and lipid metabolism [49]. Reduced lipase activity may foster lipid accumulation, exacerbating oxidative stress and potentially culminating in lipid peroxidation. This correlation underscores an intricate interplay between lipid metabolism and oxidative stress, with total oxidized values serving as a proxy for oxidative stress levels. Furthermore, the significant positive correlation between conjugated trienes and total oxidation values suggests that the presence of conjugated trienes may heighten oxidative stress.

*p*-anisidine displayed a highly significant negative correlation with lipoxygenase and a significant positive correlation with total oxidation values, suggesting that lipoxygenase activity influences lipid oxidative stability. Reduced lipoxygenase activity may render lipids more prone to oxidation, thereby elevating total oxidation values. Conversely, heightened *p*-anisidine levels may signify increased lipid oxidation, further impacting lipid stability [50].

The free fatty acid content exhibited no significant correlation with lipase and lipoxygenase but demonstrated a positive correlation with lipase and a negative correlation with lipoxygenase. This disparity may stem from lipase activity, promoting the production or release of free fatty acids, while lipoxygenase primarily participates in the oxidation of unsaturated fatty acids. It is plausible that the increase in lipoxygenase activity consumes a portion of free fatty acids or indirectly reduces free fatty acid content by modulating the lipid metabolic pathway. The intricate interactions and regulatory relationships among these enzymes and free fatty acids collectively influence the dynamic equilibrium of lipid metabolism.

## 4. Conclusions

This study offers critical insights into the biochemical changes during wheat flour maturation, particularly the effects of temperature and duration on lipid oxidation. We observed a distinct pattern in lipid metabolites, such as an initial increase in P-AV followed by a decrease and a significant rise in conjugated trienes at higher temperatures, indicative of accelerated lipid peroxidation. The TOTOX highlighted an early peak in oxidative byproducts, suggesting rapid oxidation during the initial maturation phase. The activity of key enzymes—lipase, LOX, and CAT—exhibited varied responses to maturation conditions, likely due to environmental influences on enzyme stability. Notably, an inverse correlation between lipase activity and conjugated triene formation suggests lipase’s regulatory role in lipid peroxidation. The positive correlation between P-AV and TOTOX values emphasizes the contribution of secondary lipid oxidation to overall oxidative stress. Our findings are essential for optimizing wheat flour maturation to improve quality, providing insights for the baking industry to adjust maturing conditions for specific flour attributes. Future research should focus on the long-term effects of maturation on flour quality and the impact of wheat variety on dough properties, which is vital for enhancing baking performance. This study lays a foundation for further advancements in wheat flour processing and its application in the food industry.

## Figures and Tables

**Table 1:** Pearson’s correlation of indicators during maturation of wheat flour.

Variable	FFA	K268	P-AV	TOTOX	Lipase	LOX	CAT
FFA	1						
K268	0.053	1					
P-AV	0.312	−0.266	1				
TOTOX	0.303	0.488 *	0.530 *	1			
Lipase	0.028	−0.819 **	0.199	−0.548 *	1		
LOX	−0.182	0.145	−0.582 **	−0.177	0.044	1	
CAT	0.005	−0.037	0.111	0.171	−0.066	−0.176	1

*: Correlation is significant at the 5% level. **: Correlation is significant at a 1% level.

## Data Availability

The original contributions presented in the study are included in the article, further inquiries can be directed to the corresponding author.
